# MicroED data collection and processing

**DOI:** 10.1107/S2053273315010669

**Published:** 2015-07-01

**Authors:** Johan Hattne, Francis E. Reyes, Brent L. Nannenga, Dan Shi, M. Jason de la Cruz, Andrew G. W. Leslie, Tamir Gonen

**Affiliations:** a Janelia Research Campus, Howard Hughes Medical Institute, Ashburn, VA 20147, USA; b Medical Research Council Laboratory of Molecular Biology, Cambridge, England

**Keywords:** MicroED, electron diffraction, crystallography, cryo-EM, nanocrystals

## Abstract

MicroED, a method at the intersection of X-ray crystallography and electron cryo-microscopy, has rapidly progressed by exploiting advances in both fields and has already been successfully employed to determine the atomic structures of several proteins from sub-micron-sized, three-dimensional crystals. A major limiting factor in X-ray crystallography is the requirement for large and well ordered crystals. By permitting electron diffraction patterns to be collected from much smaller crystals, or even single well ordered domains of large crystals composed of several small mosaic blocks, MicroED has the potential to overcome the limiting size requirement and enable structural studies on difficult-to-crystallize samples. This communication details the steps for sample preparation, data collection and reduction necessary to obtain refined, high-resolution, three-dimensional models by MicroED, and presents some of its unique challenges.

## Abstract 2

The collection and processing of MicroED data are presented.

## 1. Introduction   

X-ray crystallography hinges on the availability of large, well ordered crystals for accurate structure determination. The shortest side of a crystal for diffraction data collection at a regular synchrotron source is typically >50 µm in length. Particularly for difficult targets, such as membrane proteins and large protein complexes, the path from protein purification to initial crystallization hits and finally to large, well diffracting crystals may prove prohibitively resource-intensive. Furthermore, large crystals effectively preclude time-resolved studies of diffusion-triggered processes (Hajdu *et al.*, 2000 ▸). The ability to collect high-quality diffraction data from crystals smaller than 10–20 µm^3^ in volume is thus most desirable.

Both microfocus beamlines (Moukhametzianov *et al.*, 2008 ▸) and more recently X-ray free-electron lasers (XFELs) (Boutet *et al.*, 2012 ▸) allow high-resolution structure determination from such samples. In the case of microfocus beamlines, crystals with side lengths around 20 µm are routinely used for structure determination. XFELs allow data collection from crystals 1 µm and larger. Here the diffraction data are recorded, one pattern per crystal, before the crystal is destroyed by the high-powered X-ray pulse (Barty *et al.*, 2012 ▸; Chapman *et al.*, 2014 ▸). Millions of crystals are continuously streamed through the X-ray beam, giving rise to thousands of independent diffraction ‘stills’, and the data are then indexed and merged to generate a seemingly damage-free structure. More recent studies, however, suggest that radiation damage is still experienced by the sample even in XFEL experiments (Nass *et al.*, 2015 ▸). While XFELs show great promise in nano-crystallography, the high sample requirement, milligrams of protein when using liquid jets (Weierstall, 2014 ▸), coupled with limited infrastructure and high cost are currently limiting factors.

MicroED is a recently developed method in cryo-EM (electron cryo-microscopy) that allows the collection of high-resolution electron diffraction data from extremely small three-dimensional crystals that are in the range of 0.1–0.4 µm thick (Shi *et al.*, 2013 ▸) using a transmission electron microscope. Electrons are excellent probes of atomic structure because they interact more strongly with matter and deposit less energy in the crystal than X-rays (Henderson, 1995 ▸). Not surprisingly, electron diffraction has been repeatedly attempted on three-dimensional crystals over the past decades, but has until recently consistently failed to yield any refined atomic structures. Because of the traditional experimental setup, typically only a single electron diffraction pattern would be collected per crystal, but unlike in X-ray crystallography, indexing single diffraction patterns in electron microscopy is exceedingly challenging as often insufficient information is contained in a single diffraction pattern (§2.3). In 2013 we unveiled the MicroED method in which a complete diffraction tilt series was collected from a single nanocrystal, up to 90° wedge of data, allowing us to index and solve the structure of lysozyme first at 2.9 Å resolution (Shi *et al.*, 2013 ▸) and later with the improved continuous-rotation method at 2.5 Å resolution (Nannenga, Shi, Leslie & Gonen, 2014 ▸). Currently, the procedure of continuous rotation has been employed in a number of laboratories using different electron microscopes and different detectors (Nederlof, van Genderen *et al.*, 2013 ▸; Nannenga, Shi, Hattne *et al.*, 2014 ▸; Nannenga, Shi, Leslie & Gonen, 2014 ▸; Yonekura *et al.*, 2015 ▸). High-resolution structures using this method have been reported for lysozyme, catalase and the membrane protein calcium ATPase (Nannenga, Shi, Hattne *et al.*, 2014 ▸; Nannenga, Shi, Leslie & Gonen, 2014 ▸; Yonekura *et al.*, 2015 ▸; Table 1 ▸).

## 2. Methods   

While sample preparation, microscope setup and data collection likely vary from laboratory to laboratory, we detail below the protocols we employ for a successful MicroED experiment.

### 2.1. Sample preparation   

Owing to their small size, initial identification of nanocrystals suitable for MicroED can be difficult. While second-order non-linear optical imaging of chiral crystals (SONICC) (Kissick *et al.*, 2011 ▸) could provide an automated and objective means to identify small crystals, we have so far relied on visual judgment of *e.g.* the cloudiness of the drops, followed by negative-stain electron microscopy. When suitable crystals are found, about 4 µl of microcrystals in solution is dispensed onto a holey carbon or continuous carbon grid that has been cleaned in a glow-discharge device. The crystals are allowed to settle for 30 s and excess solution is removed from the grids by blotting with filter paper. Enough solution needs to be removed to minimize background contribution and allow the electron beam to penetrate the sample during subsequent exposure; however, too much blotting can dehydrate and damage the crystals. Because it can be difficult to strike the right balance between a sample that is too thick and one that has been damaged by excessive blotting, it is often beneficial to prepare several grids with a wide range of blotting times, temperatures and humidity levels.

Grids can be blotted and frozen either by hand or using an automated vitrification apparatus. Automated systems are generally preferable owing to their high reproducibility and fine control over blotting conditions, such as time and force. If the buffer is too viscous to be effectively removed, the crystals are fragile, or if too many crystals are carried off by the flow of the solution, the grids may need to be blotted manually by gently touching the backs of the grids with filter paper.

Immediately following blotting, the grids are plunged into liquid ethane. The high thermal conductivity of liquid ethane ensures the sample is frozen fast enough to prevent disruption of the crystal lattice during cooling, even in the absence of a cryo-protectant. The frozen grids are then quickly transferred into a cryo-grid box where they can be stored for long periods of time at cryogenic temperatures. Alternatively, grids may be examined in the electron microscope immediately after they are prepared. Successful blotting and freezing is a trial-and-error process and will have to be optimized individually for each sample.

#### 2.1.1. Setting up the transmission electron microscope for low-dose electron diffraction   

The alignment of the electron-optic system and the astigmatism of the lenses need to be checked and possibly corrected according to the instructions from the microscope manufacturer. The direct beam should be focused and aligned to the center of the screen and completely blocked by the beam stop. Furthermore, the grid’s *z* height should be adjusted to near the eucentric height, where the image of the sample is unaffected by the tilt of the stage on which it is mounted. A rough estimate may be found by wobbling the specimen up to 15°; the quantifoil holes, or any other identifying thin feature, should remain centered when the grid is at the eucentric height.

**Figure 1:**
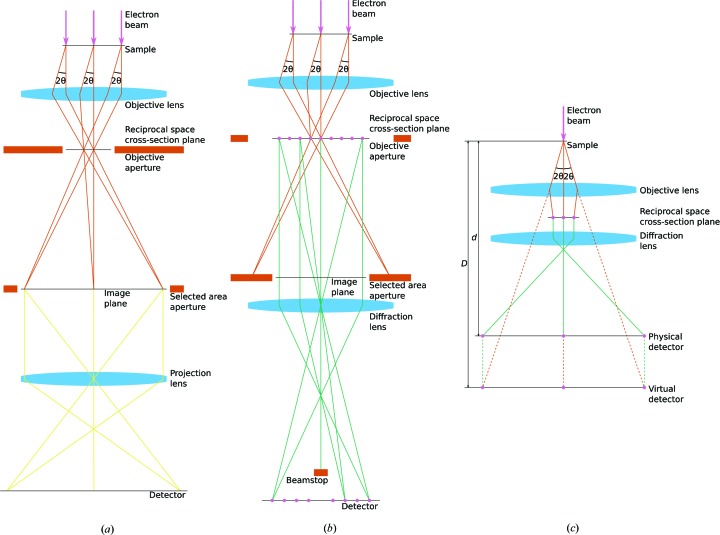
After interacting with the sample the beam (amber rays) passes through the objective lens, which forms a diffraction pattern at the cross-section plane and an image of the sample at the image plane. Only the diffraction pattern corresponding to the image of the crystal within the selected area aperture will be visible. Several rays are omitted in these simplified illustrations and the size of the image plane is exaggerated for clarity. The scattering angle (2θ) is indicated. (*a*) In bright field, the image of the crystal is magnified onto the detector (yellow rays). (*b*) In diffraction mode, the diffraction lens is positioned to form a magnified image of the diffraction pattern (green rays) on the detector. The objective aperture at the cross-section plane is fully open. (*c*) Owing to the magnification of the lenses, the distance *d* from the sample to the physical detector is typically much smaller than the distance *D* to the virtual detector. The distance to the virtual detector corresponds to the sample–detector distance in a lensless measurement using *e.g.* X-rays.

Initial grid screening is done at ultra low dose rates (<10^−6^ e Å^−2^ s^−1^) and low magnification (∼100×) in bright field (Fig. 1 ▸ *a*). In this configuration the entire grid can be quickly surveyed for the location and density of crystals and the thickness of the enveloping ice. Once crystal-containing regions are identified where the thickness of the ice, as judged by the contrast between the carbon support and the holes, is as thin as possible, the microscope is switched to over-focused diffraction mode (Fig. 1 ▸ *b*). In this configuration, individual crystals can be inspected, the *z* height fine-tuned for eucentricity, and the center spot accurately focused by minimizing the size of the spot of the direct beam at a dose rate <10^−3^ e Å^−2^ s^−1^. This should be verified using the microscope’s phosphor screen as the direct beam could otherwise damage the detector. Moreover, electron hysteresis deserves special attention so that neither the spot of the direct beam nor the image shifts when switching among the various configurations. Typically, the diffraction pattern should be recorded at a dose rate of 0.01–0.05 e Å^−2^ s^−1^, with the beam configured to be approximately 5–10 µm in diameter, the objective aperture fully open and the selected area aperture set to closely match the size of the crystal. Detailed step-by-step procedures for microscope setup were published earlier (Gonen, 2013 ▸).

#### 2.1.2. Microcrystal screening and diffraction data collection   

After finding a crystal on the grid, the crystal is centered, the eucentric height adjusted by tilting the crystal through the desired rotation range, and the selected area aperture and beam stop are inserted. An initial diffraction pattern is recorded with an exposure of 2–5 s at a dose rate of 0.01–0.05 e Å^−2^ s^−1^. If the diffraction pattern shows high-quality diffraction, a full data set is collected from that crystal. The edge of the cryo-holder limits the tilt range to approximately ±70° from the untilted orientation, in which the grid is normal to the electron beam. The combined thickness of the sample and its surrounding solvent may further restrict the useful tilt range as the amount of matter the electron beam has to traverse may become prohibitive at high tilts (Shi *et al.*, 2013 ▸). To avoid further confinement of the tilt range it should be verified that no other crystals or grid bars block the view throughout the rotation range when the selected area aperture and the beam stop are removed. If the aim of data collection is to complement an existing data set, it is often possible to approximate the crystal orientation from an initial, untilted exposure. This allows the tilt range to be optimized for measuring the desired reflections.

Initial MicroED data sets were collected as a sequence of still shots, where the crystal is held stationary during the exposure and rotated to discrete orientations only while the electron beam is blanked (Shi *et al.*, 2013 ▸). The exposure time is adjusted depending on the diffraction strength of the crystal. In this mode of data collection the TVIPS TemCam-F416 CMOS-based camera operates at its best; there is sufficient time to recharge the read-out electronics for each pixel, and consequently the signal-to-noise ratio is maximized. However, still shots introduce complications for subsequent data interpretation: the vast majority of reflections from a motionless crystal hit by an electron beam with narrow bandpass (Δ*E*/*E* ≃ 5 × 10^−6^ for a field emission gun at 200 kV) are only partially recorded. To meaningfully relate multiple observations of the same reflection to each other, the individual partial observations are either summed or converted to their full-intensity equivalent, and the accuracy of this operation decreases as the range of observed partialities increases. This issue can be overcome by oscillating the crystal during the exposure, as has long been standard practice in goniometer-based X-ray crystallography (Arndt & Wonacott, 1977 ▸). On an electron microscope crystal oscillation is complicated by difficulties in accurately positioning the stage, which is typically optimized to reduce vibrations during long exposures.

This leads to the continuous-rotation mode for MicroED data collection (Nannenga & Gonen, 2014 ▸; Nannenga, Shi, Leslie & Gonen, 2014 ▸), which captures a greater portion of each reflection because the crystal is rotated in the electron beam, but avoids absolute repositioning of the stage by moving it continuously. The stage is rotated at a constant rate where the optimal rotation rate reflects a compromise between conflicting goals, and is tuned in coordination with the exposure time (Holton & Frankel, 2010 ▸). For a given exposure time, a high rotation rate will increase the recorded fraction of each reflection on an individual frame, but a low rotation rate will ensure that even weak, high-resolution reflections accumulate enough counts on the detector before leaving their diffracting condition. Furthermore, a too high rate may result in spot overlap, while a too low rate will yield too few spots on each image.

**Figure 2:**
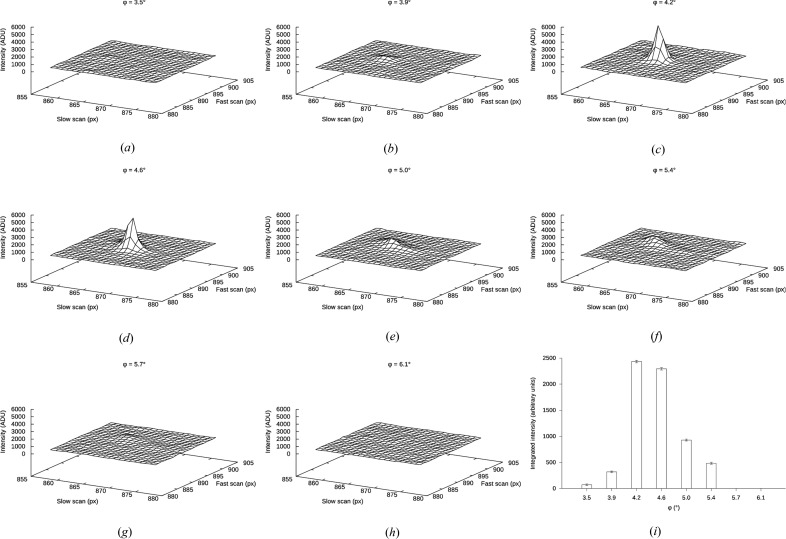
Rocking curve of the catalase (0, 10, 8) reflection at *d* = 13.7 Å, recorded in ‘rolling shutter’ mode. In all panels ϕ = 0° denotes the start of the data collection, at which point the stage is not necessarily untilted. The rotation range in all images is Δϕ = 0.36°. (*a*)–(*h*) The pixel intensities from eight successive frames as recorded by the camera, such that each node in the mesh corresponds to one pixel. (*i*) The profile-fitted intensities as integrated by *MOSFLM*, where the vertical error bars span one standard deviation. Additional rocking curves for several other spots from catalase and lysozyme are given in §3 of the supporting information.

Continuous-rotation data sets from single crystals are collected in shutterless mode in about 10 min. The detector is constantly exposed and read out at regularly spaced intervals. This mode of operation trades detector accuracy for simplified experimental setup; in particular we find that the effects of intensity accumulation and uninterrupted sample rotation during the ∼0.1 s read-out time of the detector are negligible (Fig. 2 ▸ and §2 in the supporting information).

### 2.2. Image conversion   

All MicroED measurements so far in our group have been performed using a TVIPS TemCam-F416 camera. This 16 Mpixel camera performs rudimentary image corrections internally using pre-recorded dark and gain maps, which should be selected to match the energy of the electron microscope, the exposure time and the signal strength of the sample. To satisfy the real-time constraints of the system in shutterless, or ‘rolling-shutter’, mode, the data rate is reduced by 2 × 2 binning, yielding an effective camera size of 2028 × 2048 square pixels with side length 31.2 µm. Furthermore, all synchronous read-back of any of the microscope’s dynamically changing parameters is disabled. In contrast to typical X-ray diffraction experiments at a synchrotron source (Meyer *et al.*, 2014 ▸), the user must therefore supply additional information to allow downstream processing software to reconstruct the geometry of the experiment.

(*a*) The beam center. The intersection of the direct beam with the surface of the detector plane is refined from user-defined initial values during data processing (Sauter *et al.*, 2004 ▸). With the procedure outlined in §2.1.1 above, the center of the image is a good starting point. A computational alternative, which refines the initial estimate based on the intensity variation in a single image (Baldwin & Henderson, 1984 ▸; Vonrhein *et al.*, 2011 ▸; Nederlof, van Genderen *et al.*, 2013 ▸), is given in §1 of the supporting information. This may be particularly useful if the image of the direct beam is drifting over the course of data collection because of instabilities in the electron-optical system.

(*b*) Rotation rate of the stage. While this is not directly necessary for data reduction, it is used to determine the rotation angle and range of each frame (see §2 in the supporting information for details). Because the stage of an electron microscope can generally be tilted both clockwise and counterclockwise, and the small wavelengths (§2.3) make it difficult to distinguish the handedness of the rotation, special attention needs to be paid to the sign of the rotation rate.

(*c*) The virtual sample–detector distance (Fig. 1 ▸ *c*). In principle, this can be determined from the magnification of the electron microscope, but is preferably calibrated using the ring spacing of a known powder diffraction pattern from *e.g.* gold or graphite. The circularity of the observed rings also provides a means to verify any astigmatism, which would result in a non-circular pattern. Alternatively an accurate calibration can be performed with standard crystals of known unit-cell dimensions.

We have developed conversion tools that parse a sweep of frames and output a corresponding set of diffraction images. By combining the information provided by the camera system in its output stream with information supplied by the user, these tools produce images in the Super Marty View (SMV) format, which is directly suitable for further processing in several existing data reduction packages originally developed for X-ray crystallography such as *DIALS* (Waterman *et al.*, 2013 ▸), *MOSFLM* (Leslie & Powell, 2007 ▸) and *XDS* (Kabsch, 2010*b* ▸).

Owing to limitations in the SMV format, processing programs are unaware of the specific details of the detector. The parameters below are input directly into the processing package, and can be determined from the data themselves.

(*a*) The precise interpretation of the detector gain, and therefore its estimation, depends on the downstream processing software. Typically, it is determined as the ratio of the variance and the mean of the intensities in a sufficiently large region of background pixels (Leslie, 2006 ▸; Kabsch, 2010*b* ▸). Assuming the pixels are statistically independent, processing programs can treat detector noise as the result of a Poisson process after gain-correcting the intensity values.

(*b*) The camera does not flag dead, hot or otherwise malfunctioning pixels. If their presence impairs data processing, these pixels can be discovered using *ad hoc* statistical methods outlined in §1 of the supporting information. Procedures on the TVIPS F416 camera can also help eliminate these pixels.

(*c*) To minimize radiation damage to the sample, MicroED data sets are collected in low-dose mode, and so far even the strongest low-resolution reflections have been within the linear response range of the detector. To date, there has been no need to handle overloaded pixels.

### 2.3. Diffraction geometry and indexing   

As of this writing, we are routinely indexing and integrating MicroED data for various samples using *MOSFLM/AIMLESS* (Leslie & Powell, 2007 ▸; Evans & Murshudov, 2013 ▸) and *XDS* (Kabsch, 2010*b* ▸). As these software packages are primarily developed for X-ray crystallography, it is worthwhile to keep the unique aspects of electron diffraction in mind during data reduction.

The de Broglie wavelengths used for MicroED data collection at an acceleration voltage of 200 kV are about 50× shorter than the corresponding electromagnetic wavelengths typically used for crystallographic structure determination with X-rays. Consequently, scattering angles are smaller, the Ewald sphere is less curved, and for any orientation of the crystal in the beam, the reflections in a diffracting condition fall within an almost planar wedge of reciprocal space (Nannenga & Gonen, 2014 ▸). This presents a challenge for autoindexing procedures, which rely on the periodicity in a three-dimensional space to recover both the spacing and orientation of the crystal’s lattice (Steller *et al.*, 1997 ▸; Kabsch, 2010*a* ▸; Gildea *et al.*, 2014 ▸).

In a MicroED experiment, the number of spots must be large enough for their periodic arrangement to become apparent, and their spanned volume must be big enough for the three-dimensional lattice to be determined. These requirements are generally satisfied by well diffracting crystals measured by continuous rotation, and we find that five to ten images spanning a ∼20° wedge provide sufficient information for autoindexing to work without *a priori* knowledge of the unit-cell parameters. More images may be required because, for well ordered crystals, the narrow bandpass of the electron beam (§2.1.2) leads to relatively few discernible Bragg spots on each image. If the unit cell of the crystal is known and its side lengths are unique, it is in principle possible to determine the crystal’s orientation from a single image (Jiang *et al.*, 2009 ▸) but for unknown samples the above should suffice.

Diffraction data processing generally requires accurate knowledge of the geometry of the measurement, particularly the rotation range of the sample during each exposure. In MicroED, the sample orientation is calculated during image conversion as the product of the rotation rate and the timestamp of the exposure relative to the start of the measurement (§2.2 and §2 in the supporting information). The uncertainties in both factors are relatively large, resulting in even larger inaccuracies in the derived rotation angle. Furthermore, under the assumption that the error in the timestamp is symmetrically distributed around zero, any error in the rotation rate causes the deviation of the calculated rotation angle from its true value to compound over time. For this reason it is advisable to first attempt autoindexing with several frames spaced widely enough to cover a sufficiently large wedge of reciprocal space, but recorded close enough in time such that the relative error in the rotation angle is small.

If the data reduction software fails to completely account for errors in the crystal orientation by means of the refined mis-setting angles, the residual may oftentimes be absorbed in the mosaicity. In such cases, the mosaicity acts as an error sink rather than an accurate model of lattice disorder. For small unit cells this approach may work at the price of reduced integration accuracy; for large unit cells, the ensuing spot overlap may prevent successful processing altogether.

### 2.4. Integration, scaling and merging   

The intensities in the first MicroED data sets were integrated and scaled using in-house software, which for simplicity assumed proportionality between the maximum intensity integrated for any reflection and the corresponding full intensity (Iadanza & Gonen, 2014 ▸). This worked well because the temporal intensity fluctuations in the electron beam are very small. The ability to use existing software developed for X-ray crystallography makes it straightforward to use more sophisticated integration, scaling and merging protocols. In particular, it is advisable to use the three-dimensional profile of the integrated intensities whenever a reflection is observed across multiple exposures of similar crystal orientations (Fig. 2 ▸). This profile-fitted intensity better estimates the corresponding full-intensity equivalent and helps to discriminate against spurious noise peaks. While it is difficult to obtain reliable estimates of the random and systematic errors in the measured intensities, both the merging and the refinement statistics for structures solved by MicroED (Table 1 ▸) suggest that the data quality is comparable to that obtained using conventional X-ray techniques.

In two of our studies a single nanocrystal was sufficient to allow us to collect data sets with ∼80% completeness (Table 1 ▸). For certain lattice symmetries it may, however, be difficult to collect a complete single-crystal data set because of the limited tilt range of the stage (§2.1.2). Where several isomorphous data sets are available, merging the integrated intensities from multiple crystals can generally increase the completeness. Multi-crystal merging does not necessarily increase completeness if the crystals tend to align with their crystallographic axes in similar directions (Nannenga, Shi, Hattne *et al.*, 2014 ▸; Yonekura *et al.*, 2015 ▸). In the case of bovine liver catalase, which commonly crystallizes as plates with the crystallographic *c* axis aligned perpendicular to the plane of the crystal, the limited tilt angle prevents a cone of reciprocal space from entering a diffractive condition. If the stage can only be tilted through ±α in such a case, the fraction of reciprocal space that can be observed, assuming all possible rotations around the *c* axis can be measured, is given by sin (α). In our setup, where the tilt angle is limited to ±70°, at most 94% of reciprocal space can be integrated for a system such as catalase (Glaeser *et al.*, 1989 ▸). Examples of the effects of systematically missing data on the final density in MicroED were given in Nannenga & Gonen (2014 ▸). However, for cases where the crystals do not exhibit a preferred orientation on the grid, merging data from multiple crystals can yield data sets with 100% completeness (Shi *et al.*, 2013 ▸).

### 2.5. Phasing and refinement   

All our MicroED structures to date have been phased by molecular replacement, using standard programs from X-ray crystallography (Vagin & Teplyakov, 1997 ▸; McCoy *et al.*, 2007 ▸). Subsequent manual rebuilding with interactive tools such as *Coot* (Emsley *et al.*, 2010 ▸) and refinement using standard refinement packages (Murshudov *et al.*, 2011 ▸; Afonine *et al.*, 2012 ▸) yield results of similar quality as models derived from X-ray diffraction data to the same resolution (Nannenga, Shi, Hattne *et al.*, 2014 ▸; Nannenga, Shi, Leslie & Gonen, 2014 ▸). As in X-ray crystallography, automated tools such as *BUCCANEER* or *phenix.ligand_identification* can be used to reduce the manual labor and subjective bias from visual interpretation of density maps (Cowtan, 2006 ▸; Terwilliger *et al.*, 2007 ▸). Currently *CNS* (Brunger, 2007 ▸), *Phaser*, *phenix.refine* and *REFMAC* take electron scattering factors into account during structure-factor calculation; other software may assume the diffracted intensities are due to the scattering of X-rays. At the resolution of the data sets determined by MicroED so far (Table 1 ▸), the electron scattering factors can have a noticeable impact on the refined model (Yonekura *et al.*, 2015 ▸). For molecular replacement, where the precise details of the fit of the search model to the processed data are less important, the significance of electron scattering factors is minor.

## 3. Conclusion   

MicroED builds on decades of work, both in X-ray crystallography and cryo-EM. With the recent determination of catalase and Ca^2+^-ATPase, the method matured beyond the lysozyme benchmark commonly used to evaluate new techniques in crystallography. The fundamental bottlenecks that prevented the success of electron diffraction structure solution from three-dimensional crystals have been overcome by advancements in the way in which data are collected, improvements in detector hardware and more powerful software algorithms, such that crystal structures can now be determined using a transmission electron microscope and equipment standard in most cryo-EM laboratories. The use of continuous rotation not only addresses issues with the partiality of the integrated intensities and the imperfect orientation of the stage, but appears to offset the adverse effects of diffuse and dynamic scattering (Nannenga, Shi, Leslie & Gonen, 2014 ▸). In particular, as long as the crystals are <400 nm thick, the integrated intensities are accurate enough to allow phasing by molecular replacement and subsequent atomic refinement. It is not clear where the upper limits on sample thickness lie, as there appears to be a disconnect between theory and experiment. Recent simulations on perfect lysozyme crystals suggest <100 nm to be the upper limit for refinement to *R*_work_ < 30% at 2.5 Å resolution (Subramanian *et al.*, 2015 ▸), but the authors note that effects not accounted for by the simulation may influence the estimate. Certainly, in our hands, and in the hands of other laboratories, the upper limit has been closer to 400 nm.

Depending on the quality of the microscope’s calibration there are various corrections that may need to be applied to electron diffraction images. Several aberrations (*e.g.* astigmatism, §2.2) can be corrected by calibrating the electron microscope, and for these, diagnostic tools are sufficient. Other anomalies, such as a variable rotation rate of the stage or the beam center drift, are more efficiently corrected during data analysis. Improved corrections in the analysis software, as well as investigations of the effects of instrument improvements such as energy filters, are a topic of future research, which will lead to more accurate integrated intensities.

The next frontier for MicroED appears to be experimental phasing, which relies on accurately integrated intensities and improved electron scattering tables for mapping atomic models to structure factors. While several laboratories are working on heavy metal isomorphous replacement, other strategies may also be possible. Imaging crystals followed by image processing can yield initial phase information that could then be extended by established procedures (Henderson *et al.*, 1986 ▸; Gipson *et al.*, 2011 ▸; Wisedchaisri & Gonen, 2011 ▸; Nederlof, Li *et al.*, 2013 ▸; Scherer *et al.*, 2014 ▸). Single-particle reconstructions of the particles of interest quite routinely yield a low-resolution density map that could then be used to phase the MicroED data. These avenues highlight the strengths of using a transmission electron microscope for structure determination as both phase and amplitude can be recorded accurately. As MicroED matures we expect that the method will have a long and lasting impact on the field of structural biology.

## 5. Software availability   

The source code for the image conversion software is available for download from http://cryoem.janelia.org/pages/MicroED.

## Supplementary Material

Supplementary text and figures. DOI: 10.1107/S2053273315010669/mq5031sup1.pdf

## Figures and Tables

**Table 1:** Atomic structures determined by three-dimensional electron crystallography The first four data sets were collected on a TVIPS TemCam-F416 using a field emission gun at 200kV, corresponding to a de Broglie wavelength of 0.025. Ca^2+^-ATPase and the second catalase structure were collected at 300kV (0.020) on a TVIPS TemCam-F224HD.

	Lysozyme (PDB id: 3j4g; EMDB id: EMD-2945; Shi *et al.*, 2013 ▸)	Lysozyme (PDB id: 3j6k; EMDB id: EMD-6313; Nannenga, Shi, Leslie Gonen, 2014 ▸)	Lysozyme (PDB id: 5a3e; EMDB id: EMD-6342; Nannenga, Shi, Leslie Gonen, 2014 ▸)	Catalase (PDB id: 3j7b; EMDB id: EMD-6314; Nannenga, Shi, Hattne *et al.*, 2014 ▸)	Ca^2+^-ATPase (PDB id: 3j7t; Yonekura *et al.*, 2015 ▸)	Catalase (PDB id: 3j7u; Yonekura *et al.*, 2015 ▸)
Number of crystals	3	2	1	1	99	58
Space group	*P*4_3_2_1_2	*P*4_3_2_1_2	*P*4_3_2_1_2	*P*2_1_2_1_2_1_	*C*2	*P*2_1_2_1_2_1_
Unit cell						
*a*, *b*, *c* ()	77, 77, 37	76.0, 76.0, 37.2	75.9, 75.9, 36.9	67.8, 172.1, 182.1	166.3, 64.4, 147.3	69.0, 173.5, 206.0
, , ()	90, 90, 90	90, 90, 90	90, 90, 90	90, 90, 90	90, 98.3, 90	90, 90, 90
Resolution ()†	2.9 (3.12.9)	2.5 (2.62.5)	2.5 (2.62.5)	3.2 (3.43.2)	3.40 (3.473.40)	3.20 (3.273.20)
Multiplicity	34	4.8	3.4	2.4	15.8	20.8
Completeness (%)†	92 (57)	97.2 (90.2)	80.1 (80.1)	79.4 (75.5)	67.5 (65.7)	73.0 (72.8)
*R*_work_/*R*_free_ (%)	25.5/27.8	22.0/25.5	21.3/25.3	26.2/30.8	27.7/31.5	27.2/31.7
R.m.s.d. bonds ()	0.051	0.003	0.003	0.006	0.01	0.01
R.m.s.d. angles ()	1.587	0.60	0.60	1.05	1.03	1.04

† Values in parentheses reflect the highest resolution shell.
