# Supplementary material for: MicroED data collection and processing
Source: Acta Crystallogr A Found Adv. 2015 Jul 1;71(Pt 4):353–60. doi: 10.1107/S2053273315010669 (PMC4487423; doi:10.1107/S2053273315010669)
Supplement: Supplementary file 1 [file a-71-00353-sup1.pdf]

FOUNDATIONS
ADVANCES

Volume 71 (2015)

Supporting information for article:

MicroED data collection and processing

Johan Hattne, Francis E. Reyes, Brent L. Nannenga, Dan Shi, M. Jason de la Cruz, Andrew G. W. Leslie and Tamir Gonen

1 Simple beam center refinement

Accurate knowledge of the beam center is critical for diffraction data processing (Gildea et al., 2014). At a synchrotron beamline, where the position of the direct beam is stable over long periods of time, an accurate beam center is supplied by the beamline staff, or directly inserted into the metadata of the recorded images (Meyer et al., 2014). In electron crystallography, this is generally not possible, as the intersection of the direct beam with the plane of the detector is not known to the data acquisition system, and may even drift over the course of the measurement of a single crystal due to instabilities in the electron-optical system. If the shift is sufficiently small, the beam center may be recovered by the data processing software (Sauter et al., 2004); if this is not the case, autoindexing will fail.

However, a rough estimate of the beam center is typically available. Combined with a salient feature of a diffraction pattern–the existence of a smoothly varying radial distribution function which arises from the overall intensity falloff of the background with increasing distance from the beam center–it is possible to refine the beam center on a per-image basis. The radial density distribution has been applied in image analysis of diffraction patterns before; for instance, Baldwin and Henderson (1984) used it for background subtraction in spot integration.

Given an estimated beam center $(\hat{x}, \hat{y})$, a radial average of the intensities of all the pixels in the image can be calculated as

$$\left\langle \hat{I} \right\rangle_R = \frac{1}{N_R} \sum_{x,y} I(x, y), \tag{1}$$

where $I(x, y)$ is the intensity at (x, y) and the sum runs over all pixels (x, y) such that

$$\lfloor R \rfloor \leq \sqrt{(x - \hat{x})^2 + (y - \hat{y})^2} < \lceil R \rceil. \tag{2}$$

If no pixels are rejected, the number of pixels summed, N_R, is approximately $2\pi R$. Because all image pixels at distance $\sim R$ from the beam center estimate $(\hat{x}, \hat{y})$ are considered in Eq. (1), the effect of outliers, such as pixels in Bragg spots and the shadow of the beam stop, is diminished to the point where they need generally not be rejected. Owing to the radial symmetry of the background, the intensity variance of the pixels at a distance $\sim R$ from the tentative beam center decreases as $(\hat{x}, \hat{y})$ approaches the true beam center.

It follows that

$$f = \sum_R \sum_{x,y} \left(I(x,y) - \left\langle \hat{I} \right\rangle_R \right)^2, \tag{3}$$

where the inner sum runs over the pixels that satisfy Eq. (2), has a minimum at the beam center. Given $(\hat{x}, \hat{y})$, f can be evaluated in a single pass over the pixels in an image by substituting Eq. (1) into Eq. (3). Since partial derivatives of f with respect to $\hat{x}$ and $\hat{y}$ can be readily obtained, it is amenable to optimization using e.g. conjugate gradient methods.

This method is similar to that of Nederlof et al. (2013) in that it considers all image pixels and does not rely on any previous processing of the image, such as feature extraction (Vonrhein et al., 2011) or indexing (Baldwin and Henderson, 1984). A major difference is that the method presented here does not assume any particular functional form of the spatial intensity distribution of the image. Rather, the substitution of Eq. (1) into Eq. (3) means that the radial distribution is calculated dynamically from the current beam center estimate, $(\hat{x}, \hat{y})$, as optimization progresses. It should therefore be more robust against resolution-dependent features such as solvent rings.

An excessive amount of “outlier” intensities can potentially prevent convergence to the desired minimum or may interfere with subsequent data processing. We have found that a simple rejection based on the individual intensity variance of pixels throughout a sweep is often sufficient to rescue Eq. (3): pixels with extreme variances are rejected in a second pass as either dead or hot. Similarly, pixels deviating by more than a certain threshold from their means are rejected as Bragg spots (Baldwin and Henderson, 1984).

2 Estimating the rotation angle

The stage on which the sample is mounted does not directly record its position nor its rotation range during an exposure. The data acquisition system does, however, timestamp each read-out event. Assuming the rotation rate is known and constant, $\dot{\varphi}$, the orientation of the ith frame, φ_i, relative to the orientation of the first frame, φ_0, may be calculated as

$$\varphi_i = \dot{\varphi}(t_i - t_0),$$

where t_i is the absolute time of the ith frame.

Unfortunately, the data acquisition system only records timestamps to second precision. Assuming the frames in a sweep are recorded at regular intervals, the time elapsed between two successive frames, Δt, may be obtained by solving

$$\frac{\mathrm{d}}{\mathrm{d}\Delta t}\left(\sum_i (t_i - t_0 - i\Delta t)^2\right) = 0$$

for Δt. Further assuming no frames are missing from the sweep, the solution,

$$\Delta t = \frac{\sum_i i(t_i - t_0)}{\sum_i i^2},$$

is optimal in a least-squares sense. The relative orientation of the ith frame may then be estimated as

$$\varphi_i = \dot{\varphi} i \Delta t.$$

3 Rocking curves

Example rocking curves for several strong reflections from catalase and lysozyme are shown in Figs. 1, 2, 3, 4, 5, 6, 7, and 8. Both datasets were recorded in shutterless, or “rolling shutter”, mode while the crystal was continuously rotated at 0.09°/s, and intensities were integrated using *MOSFLM*, where φ refers to the relative tilt angle of the stage. Note that in all figures $\varphi = 0°$ denotes the start of the data collection, at which point the stage is not necessarily untilted. The rotation range in all images is $\Delta\varphi = 0.36°$.

References

Joyce Baldwin and Richard Henderson. Measurement and evaluation of electron diffraction patterns from two-dimensional crystals. *Ultramicroscopy*, 14:319–335, 1984.

Richard J. Gildea, David G. Waterman, James M. Parkhurst, Danny Axford, Geoff Sutton, David I. Stuart, Nicholas K. Sauter, Gwyndaf Evans, and Graeme Winter. New methods for indexing multi-lattice diffraction data. *Acta Cryst.*, D70:2652–2666, 2014.

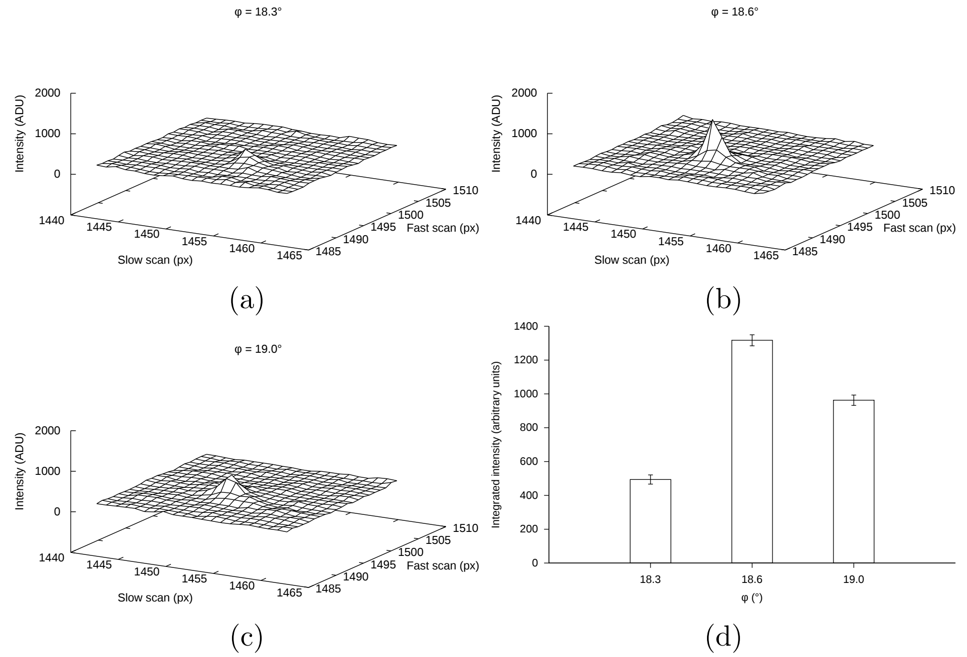

Figure 1: The $(0, 36, 13)$ reflection of catalase at $d = 4.5$ Å. Panels a, b, and c show the pixel intensities from three successive frames as recorded by the camera, such that each node in the mesh corresponds to one pixel. Panel d shows the profile-fitted intensities as integrated by *MOSFLM*, where the vertical error bars span one standard deviation.

Grischa R. Meyer, David Aragão, Nathan J. Mudie, Tom T. Caradoc-Davies, Sheena McGowan, Philip J. Bertling, David Groenewegen, Stevan M. Quenette, Charles S. Bond, Ashley M. Buckle, and Steve Androulakis. Operation of the Australian Store.Synchrotron for macromolecular crystallography. *Acta Cryst.*, D70:2510–2519, 2014.

Igor Nederlof, Eric van Genderen, Yao-Wang Li, and Jan Pieter Abrahams. A Medipix quantum area detector allows rotation electron diffraction data collection from submicrometre three-dimensional protein crystals. *Acta Cryst.*, D69:1223–1230, 2013.

Nicholas K. Sauter, Ralf W. Grosse-Kunstleve, and Paul D Adams. Robust indexing for automatic data collection. *J. Appl. Cryst.*, 37:399–409, 2004.

Clemens Vonrhein, Claus Flensburg, Peter Keller, Andrew Sharff, Oliver

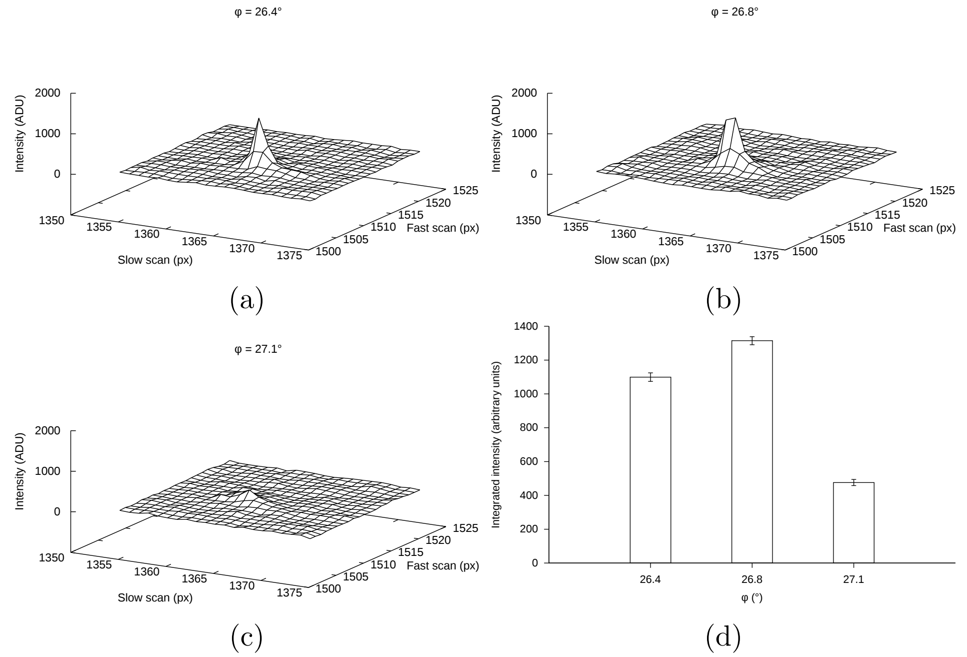

Figure 2: The $(1, 35, 6)$ reflection of catalase at $d = 4.8$ Å. Panels as in Fig. 1.

Smart, Wlodek Paciorek, Thomas Womack, and Gérard Bricogne. Data processing and analysis with the *autoPROC* toolbox. *Acta Cryst.*, D67: 293–302, 2011.

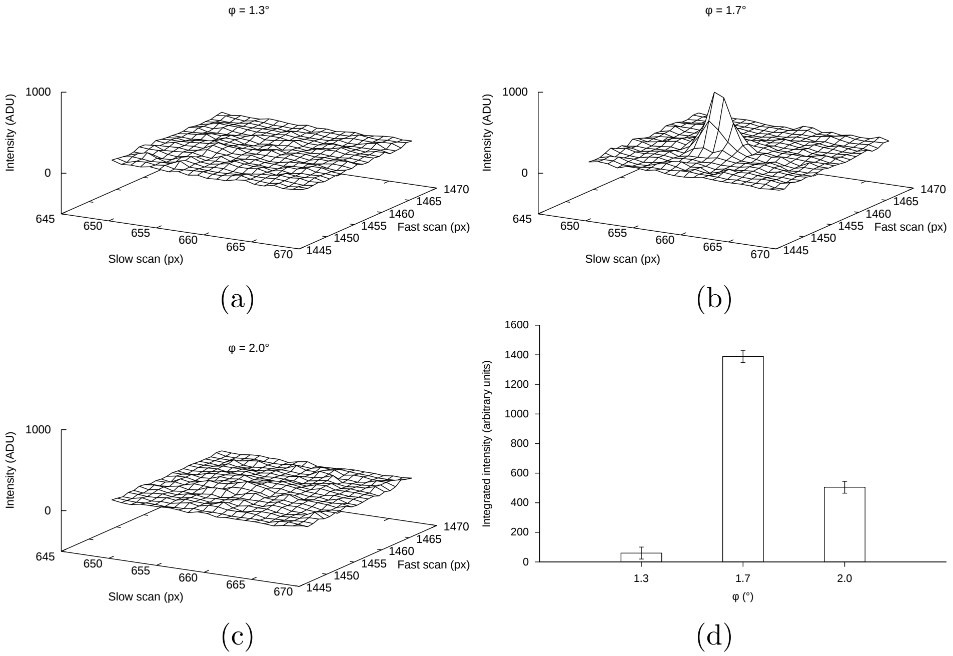

Figure 3: The $(10, 13, 20)$ reflection of catalase at $d = 5.0$ Å. Panels as in Fig. 1.

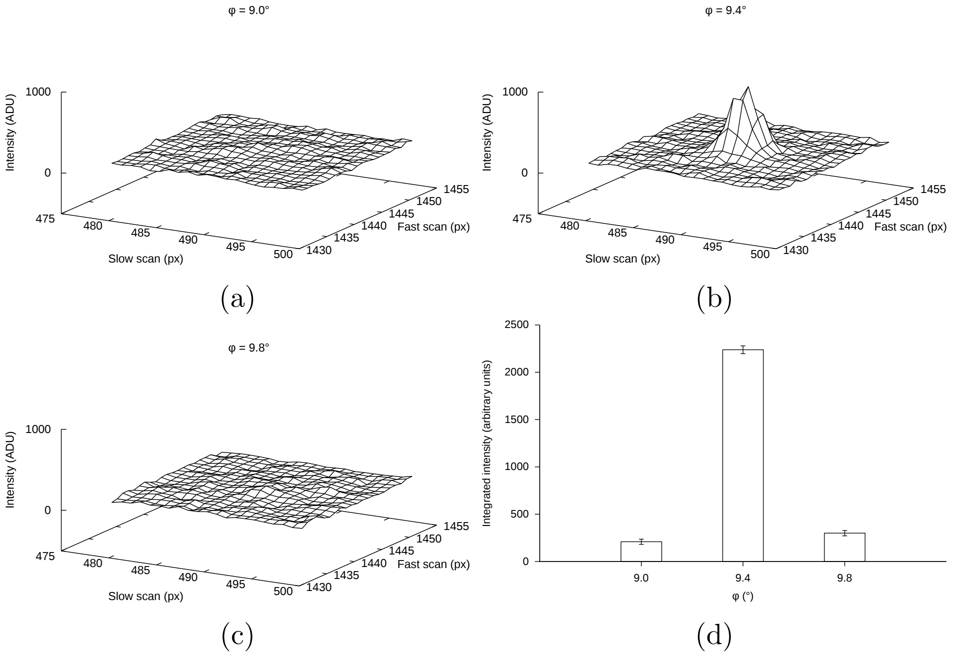

Figure 4: The $(13, 6, 25)$ reflection of catalase at $d = 4.2$ Å. Panels as in Fig. 1.

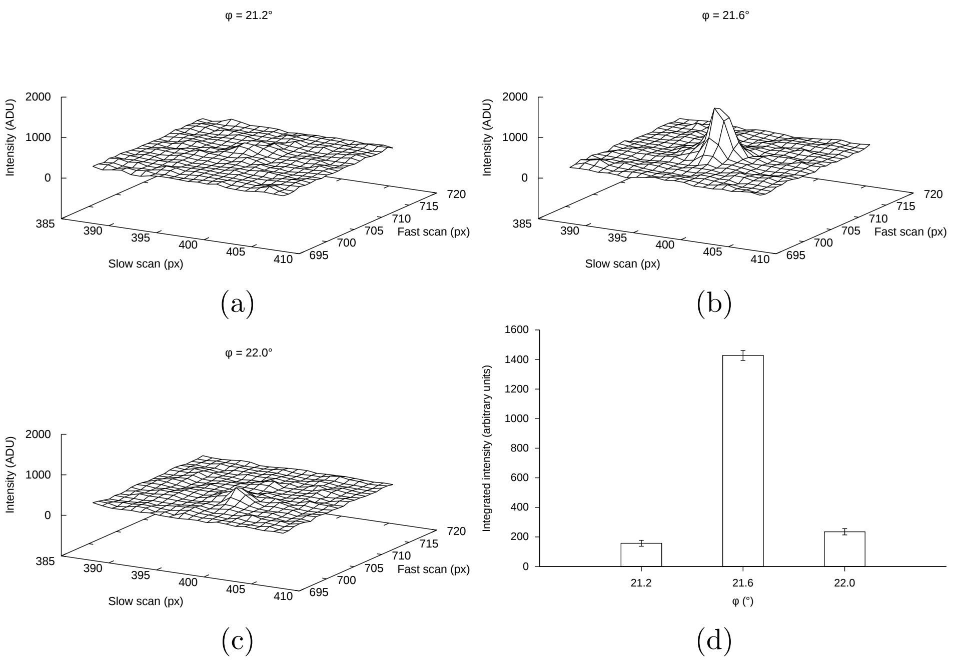

Figure 5: The $(11, 8, 6)$ reflection of lysozyme at $d = 4.2$ Å. Panels as in Fig. 1.

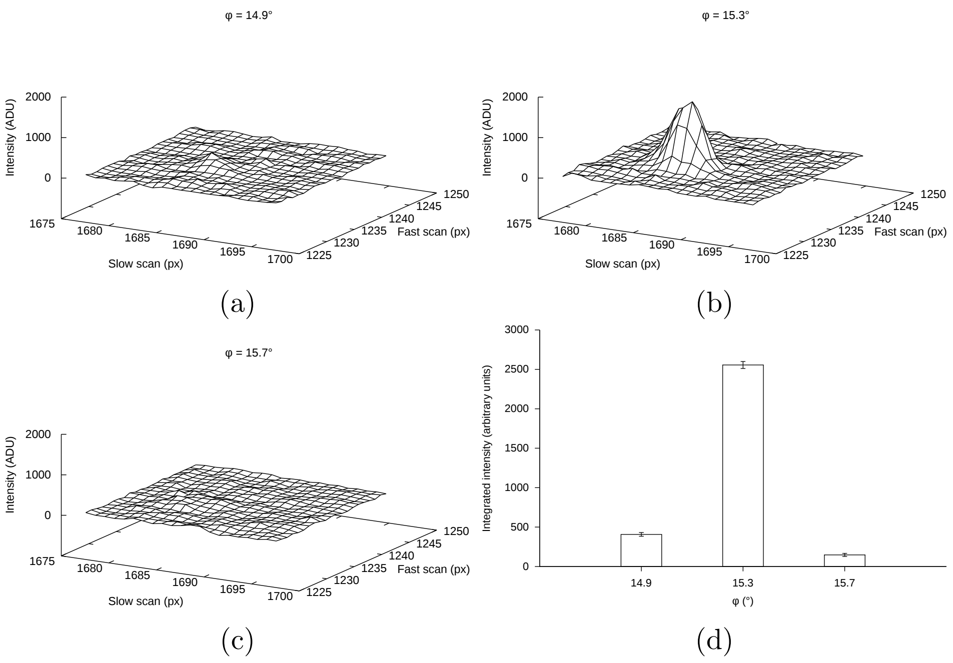

Figure 6: The $(14, 5, 6)$ reflection of lysozyme at $d = 4.0$ Å. Panels as in Fig. 1.

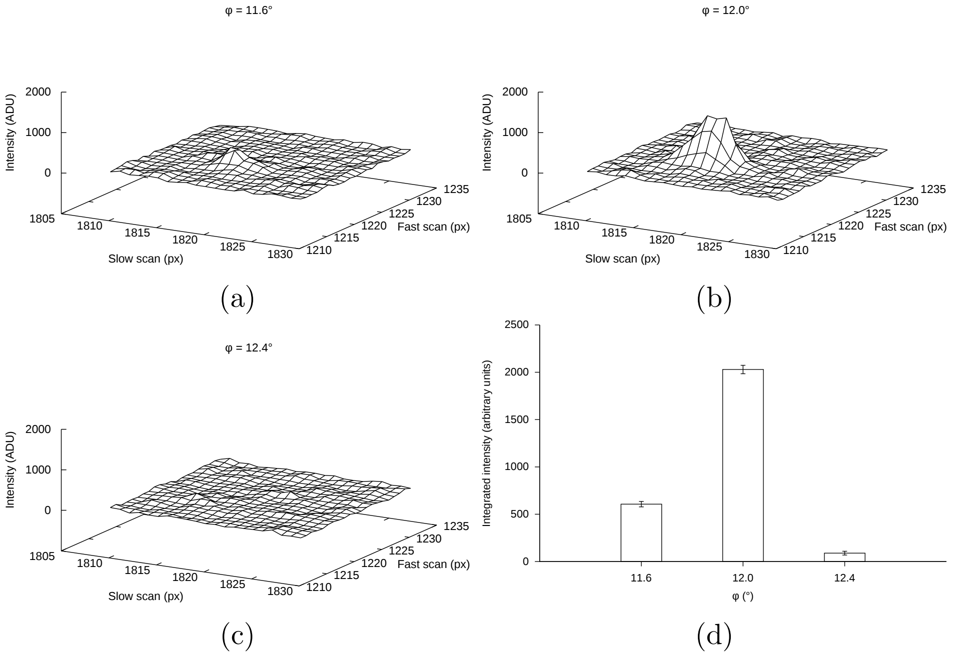

Figure 7: The $(17, 4, 7)$ reflection of lysozyme at $d = 3.4$ Å. Panels as in Fig. 1.

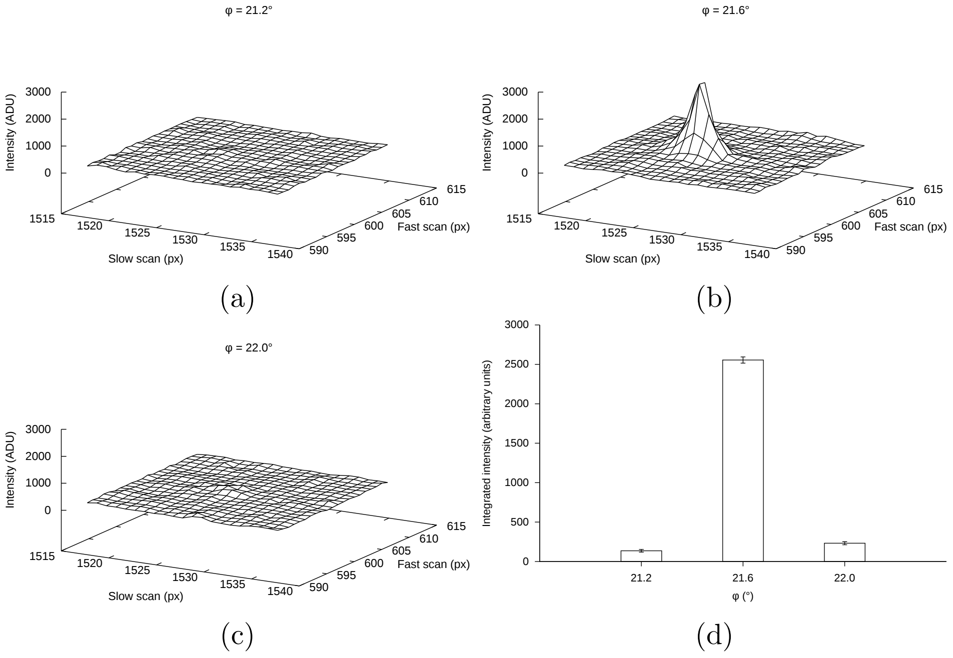

Figure 8: The $(18, 4, 1)$ reflection of lysozyme at $d = 4.2$ Å. Panels as in Fig. 1.
